# Clinical Usefulness of Red Cell Distribution Width/Albumin Ratio to Discriminate 28-Day Mortality in Critically Ill Patients with Pneumonia Receiving Invasive Mechanical Ventilation, Compared with Lacate/Albumin Ratio: A Retrospective Cohort Study

**DOI:** 10.3390/diagnostics11122344

**Published:** 2021-12-13

**Authors:** Jong Hwan Jeong, Manbong Heo, Seung Jun Lee, Yi Yeong Jeong, Jong Deog Lee, Jung-Wan Yoo

**Affiliations:** 1 Department of Internal Medicine, Gyeongsang National University Hospital, Gangnam-ro 79, Jinju 52727, Korea; Bechem6939@naver.com (J.H.J.); cough@kakao.com (M.H.); juny2278@naver.com (S.J.L.); dr202202@naver.com (Y.Y.J.); ljd8611@nate.com (J.D.L.); 2 Division of Pulmonary, Allergy and Critical Care Medicine, Department of Internal Medicine, Gyeongsang National University School Medicine, Jinju 52727, Korea

**Keywords:** red cell distribution width, albumin, lactate, pneumonia, invasive mechanical ventilation, mortality

## Abstract

The value of the red cell distribution width (RDW) is associated with prognosis in critically ill patients. A simplex combined index—the RDW/albumin ratio—has been proposed for the prediction of mortality, as has the lactate/albumin ratio. The aim of this study was to evaluate the clinical utility of the RDW/albumin ratio regarding 28-day mortality in critically ill patients with pneumonia. Clinical data of critically ill patients with pneumonia who were hospitalized in the medical intensive care unit from May 2018 to December 2020, and received invasive mechanical ventilation (IMV), were reviewed retrospectively. The values of RDW, lactate, and albumin measured at the time of IMV, were used for the index calculations. Of the 234 patients, the median age was 76 years, and 74.2% were male. The 28-day mortality rate was 47.3%. The median RDW/albumin ratio was significantly higher in non-survivors than survivors at 28 days (5.8 vs. 4.9, *p* < 0.001). A higher RDW/albumin ratio was significantly associated with increased 28-day mortality (odds ratio [OR] 1.338, 95% confidence interval [CI] 1.094–1.637, *p* = 0.005). The area under the receiver operating curve (AUROC) was 0.694 (95% CI: 0.630–758, *p* < 0.005) to discern 28-day mortality without significant difference, compared with that of the lactate/albumin ratio. Our data suggest that high RDW/albumin ratio has a similar predictability to the lactate/albumin ratio in critically ill patients with pneumonia receiving IMV.

## 1. Introduction

Pneumonia is a common infection of the lower respiratory tract, and contributes to a substantial economic healthcare burden [1,2]. It is caused by diverse microbes including bacteria, viruses or fungi [3], and commonly accompanied by severe hemodynamically and respiratory-compromised conditions [4,5]. These devastating complications generally make intensive care unit (ICU) admission inevitable for performing life-saving interventions, such as fluids, vasopressor infusion, or invasive mechanical ventilation and so forth [6,7]. Despite this intensive care, the mortality rate in these conditions remains high, ranging from 10% to 50% [5,8,9,10].

Inflammation induced by host-pathogen interaction is the major pathogenesis for severe conditions secondary to pneumonia [11,12,13]. Therefore, it is important to ameliorate inflammation in tandem with treatment of causative pathogens using optimal antiviral, bacterial, or fungal agents. It is important to identify or repurpose new or known inflammatory single or combined indexes that can predict the clinical outcomes of critically ill patients with pneumonia, hospitalized in the ICU, because lung inflammation is a major contributor to adverse outcomes [14,15].

Red cell distribution width (RDW) is easily measured from venous blood and routinely shown in complete blood count profiles. RDW is a marker to reflect the variability in red blood cell size and its increase is called anisocytosis. Several conditions such as inflammatory reactions are associated with increased RDW [16]. The RDW has been related to outcomes of various clinical conditions [17,18,19,20], as well as in critical illness [21]. Elaborated mechanisms for increased RDW in critically ill conditions, including severe pneumonia or sepsis, remain to be determined, but inflammation increases RDW value by stimulating production of circulating premature erythrocytes from bone marrow [22]. Furthermore, excessive reactive oxygen species (ROS) under oxidative stress also stimulate erythropoiesis, leading to anisocytosis and increased RDW [23]. There are a few studies showing that the combination of RDW and other feasible markers in clinical practices is more accurate for predicting mortality than RDW alone.

Albumin is a vital protein to modulate oncotic pressure [24], and a previous study demonstrated that its concentration correlated with the permeability of pulmonary vessels in critically ill patients [25]. Furthermore, several studies have revealed that low serum albumin concentration had the effect on increased mortality in those with pneumonia, sepsis or cancer [26,27,28,29,30]. Arterial lactate/albumin ratio, combined index, is associated with mortality in critically ill patients with sepsis or septic shock, in some studies [31,32,33].

A recent study showed that the albumin-RDW score is the independent factor of 90-day mortality in patients with severe community-acquired pneumonia [34]. The clinical relevance of the RDW/albumin ratio in critically ill patients with pneumonia that underwent invasive mechanical ventilation (IMV) still remains to be fully elucidated. In addition, this ratio has not been compared with other combined indexes used to predict mortality.

This study aimed to assess the relationship between the RDW/albumin ratio and 28-day mortality and compare this ratio with the lactate/albumin ratio.

## 2. Materials and Methods

### 2.1. Patients

Medical records of critically ill patients with pneumonia hospitalized in the medical ICU at a university-affiliated hospital from May 2018 to December 2020 were reviewed, retrospectively. Patients less than 18 years old, with active malignancy, and those who received only non-invasive oxygen therapy, such as high flow nasal cannula oxygen therapy during ICU admission, were excluded for final analysis.

### 2.2. Data Collection

Baseline characteristics such age, gender, age, body mass index (BMI), and underlying diseases upon ICU admission were obtained. Severity of illness and organ dysfunction at ICU admission were assessed by calculating acute physiology and chronic health evaluation (APACHE) II and sequential organ failure assessment (SOFA) scores. Clinical characteristics including complications and treatment modalities were collected. Laboratory results (white blood cell, hemoglobin, RDW, platelet, D-dimer, urea, albumin, C-reactive protein [CRP], and lactate) were collected. CURB-65 index was calculated to present the severity of pneumonia.

### 2.3. Study Outcome and Definition

The 28-day mortality rate was the main clinical outcome for this study. Septic shock was defined using sepsis III diagnostic criteria [6], and ARDS met the Berlin definition [35].

The RDW/albumin ratio was produced based on the time of MICU admission and the application of invasive mechanical ventilation. The formula was as follows: RDW (%) divided by albumin concentration (g/dL). The lactate/albumin ratio was also calculated; lactate (mmol/L) divided by albumin concentration (g/dL).

### 2.4. Statistical Analysis

Continuous data were presented as the median with interquartile range (IQR) and the Mann–Whitney U test was used for comparison. Non-continuous data were reported as numbers (%) and were compared using Fisher’s exact test and/or the chi-square test. By using a receiver-operating characteristic (ROC) curve and the Youden method, a cut-off value was determined to classify low and high RDW/albumin ratios [36]. Factors associated with 28-day mortality were determined using multiple logistic regression analysis. Factors with significance in univariate analysis were entered in a multivariate analysis using backward stepwise methods. The Kaplan–Meier method and the log-rank test were used to compare the 28-day mortality between low and high RDW/albumin ratio groups. A *p*-value less than 0.05 was considered statistically significant. All analysis of data were performed using SPSS software version 22.0 (IBM Corp., Armonk, NY, USA) and MedCalc^®^ for Windows, Version 15.22.4 (MedCalc^®^ Software, Ostend, Belgium) and figures were generated using Prism 5.01 (GraphPad Software Inc., San Diego, CA, USA).

## 3. Results

### 3.1. Characteristics of the Patients

The median age of 234 patients was 76 years, and 73.1% were men. The 28-day mortality rate was 46.6%. The baseline and clinical characteristics between survivors and non-survivors at 28-days are shown in Table 1. The proportions of male patients and those with chronic liver disease were significantly higher in non-survivors than in survivors. APACHE II and SOFA scores calculated at MICU admission were significantly higher in non-survivors than survivors at 28 days. Clinical presentations of septic shock, ARDS, and acute kidney injury more frequently developed in non-survivors than in survivors.

Laboratory parameters and their comparisons are shown in Table 2. Concentrations of hemoglobin and albumin were significantly lower in non-survivors than survivors, whereas RDW, platelets, and arterial lactate values were higher. The partial pressure of the oxygen/fractioned inspired oxygen ratio, reflecting oxygenation, was significantly lower in non-survivors than in survivors.

### 3.2. RDW/Albumin Ratio and Factors Related to a 28-Day Mortality

The median RDW/albumin ratio of all patients was 5.2 [interquartile range (IQR), 4.3–6.4]. Non-survivors had a significantly higher median RDW/albumin ratio than survivors [5.8 (IQR, 4.8–7.1) vs. 4.8 (IQR, 3.9–5.6), *p* < 0.001].

Univariate and multivariate logistic analyses for evaluation of factors related to a 28-day mortality are presented in Table 3. In the univariate analysis, the male gender, chronic liver disease, APACHE II and SOFA score, ARDS, hemoglobin, platelet, lactate, partial pressure of the oxygen/fractioned inspired oxygen ratio and the RDW/albumin ratio were significantly associated with a 28-day mortality. In the multivariate analysis, a high RDW/albumin ratio (odds ratio [OR] 1.379, 95% confidence interval [CI] 1.103–1.723, *p* = 0.005) was associated with 28-day mortality, along with old age, high SOFA score, and lower partial pressure of oxygen/fractioned inspired oxygen ratio.

### 3.3. Determination of the Cut-Off Value for RDW/Albumin Ratio, and the Comparison with Lactate/Albumin Ratio for Discrimination of 28-Day Mortality

**Figure 1:**
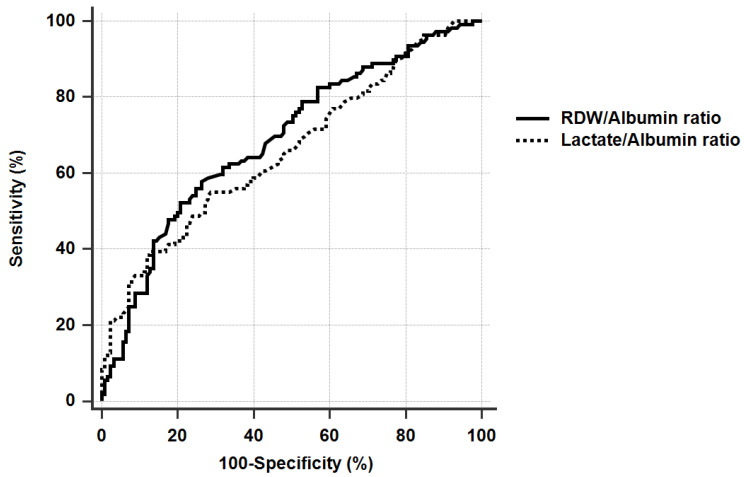
The comparison of the AUROCs. (RDW/Albumin ratio, 0.694 and lactate/albumin ratio, 0.664, *p* = 0.455).

**Figure 2:**
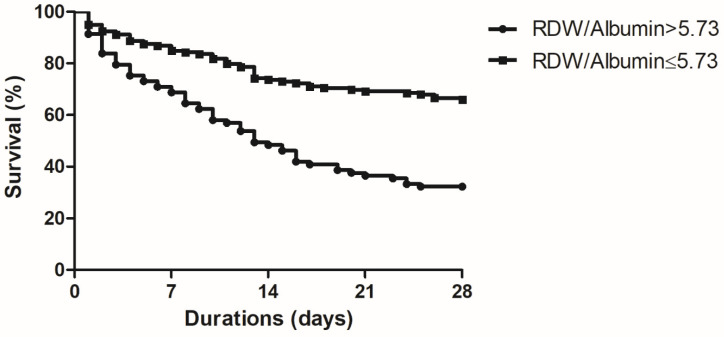
Kaplan–Meier survival curve at 28 days, between non-survivors and survivors with pneumonia receiving invasive mechanical ventilation (*p* < 0.001).

The cut-off value for the RDW/albumin ratio was 5.73 (52.3% sensitivity and 79.2% specificity) to discriminate 28-day mortality, and the area under the ROC (AUROC) curve was 0.688 (95% CI: 0.625–0.747, *p* < 0.001). The cut-off value for the lactate/albumin ratio was 1.92 (39.4% sensitivity and 87.2% specificity) to discern 28-day mortality, and the AUROC was 0.658 (95% CI: 0.593–0.719, *p* < 0.001) (Figure 1). There was no difference in the ability of the RDW/albumin and lactate/albumin ratios in predicting 28-day mortality (*p* = 0.486). When the two groups were divided according to the RDW/albumin ratio cut-off, patients with a ratio >5.73 had a higher survival rate (Figure 2, *p* < 0.001).

## 4. Discussion

The present study revealed that the median RDW/albumin ratio was significantly higher in critically ill patients with pneumonia who died at 28 days after receiving IMV. The increased RDW/albumin ratio was associated with 28-day mortality in the multivariate analysis, which suggests that a high RDW/albumin ratio may be a potential predictor of 28-day mortality in these patients. Furthermore, the RDW/albumin ratio was comparable to the lactate/albumin ratio in predicting 28-day mortality. Patients with an RDW/albumin >5.73 had a significantly higher 28-day mortality rate than those with an RDW/albumin ratio ≤5.73.

Pneumonia is a commonly encountered lower respiratory tract disease, and contributes considerably to hospitalization and health burdens [1,2]. Severe conditions such as septic shock or acute respiratory failure develop [4,5,6,33], and these conditions typically require ICU admission and intensive management including fluid, vasopressor infusion or mechanical ventilation [7,37]. Inflammation is initiated by the host-pathogen interaction, and is a major pathogenesis for patients to develop fatal outcomes [11,12,13]. Therefore, the use of agents to eradicate the pathogens and the recognition and control of inflammation is crucial to improve clinical outcomes in critically ill patients with pneumonia who are receiving IMV. Based on the inflammatory response as a major mechanism, identification of new inflammatory markers or repurposing of the previously known indices could be valuable to determine severity or predict clinical outcomes [14,15].

The RDW expresses the variation of red blood cell size, and is known to increase in response to inflammatory stimuli [16]. Increased RDW has been associated with poor outcomes in many clinical situations, such as pneumonia [38], sepsis/septic shock [19], ARDS [39], cardiovascular diseases [40], surgery [41], and malignant conditions [18]. RDW is a marker to reflect the variability in red blood cell size and its increase is called anisocytosis. Several conditions, such as inflammatory reaction, are associated with increased RDW [16]. The RDW has been related to outcomes in various clinical conditions [17,18,19,20], including critical illness [21]. Elaborated mechanisms for increased RDW in critically ill conditions, including severe pneumonia or sepsis, remain to be determined; inflammation is induced to produce the circulation of premature erythrocytes from the bone marrow [22]. In addition, oxidative stress produces reactive oxygen species (ROS) excessively, which also stimulate erythropoiesis, leading to anisocytosis and increased RDW [23]. There are a few studies that suggest the combination of RDW and other feasible markers in clinical practice will predict mortality more efficiently than RDW alone.

Serum albumin is a laboratory value, routinely measured from blood in hospitalized patients; it plays many roles, including in acute phase reactions [42] and in the control of oncotic pressure [24,25]. Furthermore, several studies have indicated that that its concentration was a prognostic factor in patients with pneumonia or sepsis [26,28,29,30]. As therapeutic role, in SAFE study, the administration of albumin has similar outcomes compared to saline in the ICU. Furthermore, in this study, patients receiving albumin had less fluid balance than those receiving saline [43]. Other studies have shown that the replacement of albumin in hypoalbumic critically ill patients reduced the severity of organ dysfunction [44].

In a post hoc anslysis of ALBIOS study, albumin replacement added to crystalloid showed more hemodynamic stability than crystalloid alone, in patients with severe sepsis and septic shock [45].

New combined indexes have been developed and investigated for clinical impact on diagnostic or prognostic markers in critically ill patients. The lactate/albumin ratio was reported to be correlated with short-term mortality in patients with sepsis or septic shock [31,33,46]. Lactate is typically measured from arterial blood to predict severity, prognosis, or for treatment monitoring in cases of sepsis/septic shock. However, the relationship between venous and arterial lactate remains controversial [47,48,49], and it could be difficult to take arterial blood continuously, due to the risk of peripheral arteries in critical care practices. Consequently, it is not feasible to calculate the lactate/albumin ratio for all critically ill patients.

Considering the feasible measurement from venous blood and the clinical role of RDW on prognosis demonstrated by many studies, the RDW/albumin ratio was applied to evaluate its ability to predict mortality in patients with pneumonia receiving IMV. In the previous study of patients with ARDS, RDW/albumin was associated with 60-day mortality [50]. Chen et al. reported that the albumin-RDW score derived from the ROC curve was the independent risk factor of 90-day mortality in patients with severe community-acquired pneumonia [34]. This study supports the usefulness of the combined index of RDW and albumin in pneumonia.

The present study showed that high RDW/albumin was associated with increased 28-day mortality in patients with pneumonia. This result suggests that RDW/albumin plays a role in a new index of predicting mortality in patients with pneumonia receiving IMV. In addition, there was no significant difference between the RDW/albumin and lactate/albumin ratio, in predicting 28-day mortality. This finding suggests that the RDW/albumin ratio may be as useful as the lactate/albumin ratio.

There are several limitations in this study. First, considering the retrospective design conducted in a single center, and the limitation of the sampling analysis, selection and sampling bias cannot be excluded. Therefore, the results should be interpreted cautiously when applied in other clinical settings. Accurate analysis of sample size in the future prospective study is needed to overcome the limitations of our results. Second, the RDW/albumin ratio at a single time point was calculated, and therefore the clinical implication of later time-points cannot be excluded. Whether changes in the RDW/albumin ratio will impact the mortality of these patients requires further studies.

In conclusion, RDW/albumin ratio is easily calculated in clinical practice. A high RDW/albumin ratio in critically ill patients with pneumonia receiving IMV is associated with 28-day mortality, and there is a similar predictability level for the lactate/albumin ratio, which is a useful combined index of critical illness. The RDW/albumin ratio has the potential of a repurposed index to predict the outcome of critically ill patients with pneumonia receiving invasive mechanical ventilation in the ICU.

## Figures and Tables

**Table 1:** Baseline characteristics of total patients, survivor and non-survivors at 28-day.

Variables	Total	Survivor	Non-Survivors	*p*-Value
	N = 234	N = 125	N = 109	
Age, years old	76 (64.8–81)	76 (61.5–80)	76 (65.5–81)	0.323
Gender, male	171 (73.1)	83 (66.4)	88 (80.7)	0.014
BMI, (kg/m^2^)	20.9 (18.5–23.3)	21.3 (18.3–23.7)	20.8 (18.6–23)	0.684
Diabetes mellitus	95 (40.6)	47 (37.6)	48 (44)	0.317
Chronic kidney disease	31 (12.5)	16 (12.8)	15 (13.8)	0.829
Chronic heart failure	25 (10.7)	13 (10.4)	12 (11)	0.880
Chronic liver disease	28 (12)	9 (7.2)	19 (17.4)	0.016
Cerebrovascular disease	49 (20.9)	28 (22.4)	21 (19.3)	0.557
COPD	43 (18.4)	27 (21.6)	16 (14.7)	0.173
ILD	12 (5.1)	5 (4)	7 (6.4)	0.402
APACHE II	24 (19–28)	22 (17–25)	27 (22–33)	<0.001
SOFA	11 (8–13)	9 (7–11)	13 (10–15)	<0.001
Types of pneumonia				
CAP	117 (50)	65 (52)	52 (47.7)	0.512
Nosocomial pneumonia	117 (50)	60 (48.1)	57 (52.3)	
Septic shock	178 (76.1)	85 (68)	93 (85.3)	0.002
ARDS	131 (56)	58 (46.4)	73 (67)	0.002
AKI	117 (50)	46 (36.8)	71 (65.1)	<0.001
HFNC before IMV	77 (32.9)	28 (22.4)	49 (45)	<0.001
RRT	54 (23.1)	14 (11.2)	40 (36.7)	<0.001
Prone position	15 (6.4)	6 (4.8)	9 (8.3)	0.282
ECMO	10 (4.3)	5 (4)	5 (4.6)	0.825

BMI, body mass index; COPD, chronic obstructive pulmonary disease; ILD, interstitial lung disease; APACHE, acute physiology and chronic health evaluation; SOFA, sequential organ failure assessment; ARDS, acute respiratory distress syndrome; AKI, acute kidney injury; HFNC, high-flow nasal cannula oxygen therapy; IMV, invasive mechanical ventilation; RRT, renal replacement therapy; ECMO, extracorporeal membrane oxygenation.

**Table 2:** Comparisons of laboratory and ventilator values at intubation and mechanical ventilation between survivors and non-survivors at 28 days.

Variables	Total	Survivors	Non-Survivors	*p*-Value
	N = 234	N = 125	N = 109	
WBC, · 10^3^/mm^3^	13.6 (6.6–20.5)	13.2 (8–19.1)	14.1 (5.5–21.9)	0.994
Hb, g/dL	10.9 (9.4–12.2)	11.2 (9.9–12.5)	10.5 (8.9–11.9)	0.007
RDW, %	14.4 (13.4–15.6)	14 (13.2–15.2)	14.9 (13.6–16.2)	0.008
Platelet, · 10^3^/mm^3^	181 (106–248)	190 (143–264)	146 (83.5–225.5)	0.001
D-dimer, ug/mL (n = 175)	3.6 (1.7–7.3)	3.4 (1.6–6.4) [90/125]	3.7 (1.9–8.4) [85/109]	0.195
Urea, mg/dL	26.9 (17.9–42.5)	23.6 (15.9–39)	30.7 (21.4–45)	0.03
Albumin, g/dL	2.8 (2.3–3.2)	3 (2.6–3.5)	2.6 (2.2–3)	<0.001
CRP, mg/dL	15.9 (9.1–25.9)	14.4 (7.9–25.9)	18.8 (11–26)	0.141
Lactate,	3 (1.8–5.2)	2.6 (1.6–4.1)	3.6 (2–6.5)	0.002
P/F ratio	140 (102–198.4)	161.7 (125.7–226.9)	122.9 (93.2–171.7)	<0.001
CURB-65 score	4 (4–5)	4 (3–5)	5 (4–5)	<0.001
≥4	188 (80.3)	90 (72)	98 (89.9)	0.001

WBC, white blood cell; Hb, hemoglobin; RDW, red cell distribution width; CRP, C-reactive protein; PaCO2; P/F, partial pressure of oxygen/fractioned inspired oxygen.

**Table 3:** Univariate and multivariate analysis for factors associated with 28-day mortality.

	Univariate			Multivariate		
Variable	OR	95% CI	*p*-Value	OR	95% CI	*p*-Value
Age	1.018	0.997–1.039	0.086	1.046	1.017–1.076	0.002
Male gender	2.120	1.160–3.878	0.015	2.015	0.955–4.248	0.066
CLD	2.721	1.175–6.300	0.019	-	-	-
APACHEII	1.150	1.098–1.205	<0.001	-	-	-
SOFA	1.340	1.220–1.471	<0.001	1.280	1.151–1.424	<0.001
ARDS	2.342	1.376–3.987	0.002	1.931	0.981–3.798	0.057
Hb	0.848	0.746–0.964	0.012	0.939	0.813–1.085	0.394
Platelet	0.996	0.994–0.999	0.002	-	-	-
Lactate	1.187	1.084–1.299	<0.001	-	-	-
P/F ratio	0.991	0.987–0.995	<0.001	0.993	0.988–0.999	0.03
RDW/Albumin ratio	1.545	1.282–1.862	<0.001	1.379	1.103–1.723	0.005

OR, odds ratio; CI, confidence interval; CLD, chronic liver disease; APACHE, acute physiology and chronic health evaluation; SOFA, sequential organ failure assessment; ARDS, acute respiratory distress syndrome; Hb, hemoglobin; P/F, partial pressure of oxygen/fractioned inspired oxygen. RDW, red cell distribution width.

## Data Availability

Data are available upon reasonable request from the corresponding author.
